# Report from the OECI Oncology Days 2014

**DOI:** 10.3332/ecancer.2014.496

**Published:** 2014-12-31

**Authors:** WH van Harten, G Stanta, G Bussolati, P Riegman, G Hoefler, KF Becker, G Folprecht, M Truini, J Haybaeck, R Buiga, M Dono, A Bagg, JA López Guerrero, S Zupo, F Lemare, F de Lorenzo, N Goedbloed, D Razavi, J Lövey, PA Cadariu, GA Rollandi, F Paparo, M Pierotti, T Ciuleanu, P De Paoli, G Weiner, M Saghatchian, Claudio Lombardo

**Affiliations:** 1 The Netherlands Cancer Institute, Amsterdam,The Netherlands; 2 Department of Medical Sciences, University of Trieste, Trieste, Italy; 3 Department of Medical Sciences, University of Torino, Torino, Italy; 4 Erasmus Medical Centre, Rotterdam, The Netherlands; 5 Johannes Haybaeck, Institute of Pathology, Medical University of Graz, Graz, Austria; 6 Institute of Pathology, Technische Universität München, München, Germany; 7 University Cancer Centre, University Hospital Carl Gustav Carus, Dresden, Germany; 8 IRCCS AOU San Martino/IST National Cancer Institute of Genoa, Genoa, Italy; 9 The Oncology Institute “Prof. Dr. Ion Chircuţă”, Cluj-Napoca, Romania; 10 IRCCS AOU San Martino/IST, National Cancer Institute of Genoa, Italy; 11 Hematology, University of Pennsylvania, PA, USA; 12 Fundación Instituto Valenciano de Oncología, València, Spain; 13 Clinical Pharmacy Department, Gustave Roussy Comprehensive Cancer Centre, Villejuif, France; 14 European Cancer Patient Coalition, Brussels, Belgium; 15 Institut Jules Bordet et Université Libre de Bruxelles, Clinique de Psycho-Oncologie et des Soins Supportifs, Brussels, Belgium; 16 National Institute of Oncology, Budapest, Hungary; 17 Department of Radiology, E.O. Ospedali Galliera, Mura della Cappuccine, Genoa, Italy; 18 IRCCS Fondazione Istituto Nazionale Tumori Milan, Milan, Italy; 19 Centro di Riferimento Oncologico, IRCCS, Aviano, Italy; 20 University of Iowa, IA, USA; 21 Medical Oncology Department, Gustave Roussy Comprehensive Cancer Centre, Villejuif, France; 22 Organisation of the European Cancer Institutes, C/o SOS Europe, Genoa, Italy

**Keywords:** comprehensive cancer centres, accreditation, designation, personalized medicine, organisation

## Abstract

The 2014 OECI Oncology Days was held at the ‘Prof. Dr. Ion Chiricuta’ Oncology Institute in Cluj, Romania, from 12 to 13 June. The focus of this year’s gathering was on developments in personalised medicine and other treatment advances which have made the cost of cancer care too high for many regions throughout Europe.

## Introduction

The 2014 OECI Oncology Days, organised in conjunction with the 36th OECI General Assembly and the 85th Anniversary of the ‘Prof. Dr. Ion Chiricuta’ Oncology Institute, aimed to debate crucial aspects of a modern approach to cancer care, giving more options to European patients to receive the best cures and taking into account the sustainability of the application of new therapeutic approaches.

Personalised medicine has the potential to transform the way health care is delivered by tailoring solutions to the individual patient and gaining in efficiency and efficacy. However, budget pressure and lack of professional skills in molecular diagnosis, are preventing patients and health systems from gaining access to some of these new approaches. The governments across Europe must therefore be invited to adapt their health systems, to avoid disparities in cancer care without increasing the burden on society.

As cancer health care costs represent 5–10% of the total health care costs in most European countries, the restructuring of the hospital care landscape that is taking place in many Western countries will be a challenge to those countries which are now in a development stage of their health care systems.

The decision to hold the Oncology Days 2014 in Cluj-Napoca clearly reflects the awareness that the OECI has to improve the development of politics and programmes to involve our Eastern colleagues more. This conference reflects the OECI commitment to enhance the comprehensiveness and internal organisation of the cancer centre as a model to better support the quality and the sustainability of cancer care without disparities amongst countries, regions, and social provenance.

This report is a brief overview of the speakers and topics discussed during the meeting.

## Retrospective survival studies, Giorgio Stanta

There is a lot of criticism in the literature regarding clinical retrospective studies, as well as the long time usually required for validation of clinical biomarkers. The problem with these studies, partially in common with prospective studies as well, is the selection of tissues and their microdissection related to the high level of heterogeneity usually found in tumours. This is also due to the lack of standardisation of the molecular methods used in archiving tissues, starting from extraction of nucleic acids. Only with a correct microdissection and with stardardised methodologies, it is possible to obtain reproducible results that can be informative for clinical development. These archive tissues will be increasingly important in the future to better subgroup therapy patients, to recognise new acquired resistance biomarker and to establish the real efficacy of the new therapies.

Today the major European organisations are starting to improve the quality of these studies with specific working groups and an increase in collaboration between the European biobanking infrastructure (BBMRI-ERIC), the European Cancer Institutes (OECI), oncologists (ESMO) and pathologists (ESP). Therefore, a workshop was recently organised in Graz to start an international collaboration amongst European organisations to improve the quality and reduce the time needed for this type of clinical study. A White Document is being prepared and several working groups are standardising pre-analytical conditions, methods and microdissection of tissues. Different models of study design are in development for clinical research, verification and validation of biomarkers.

## Pre-analytical conditions in tissues, Gianni Bussolati

Histopathological diagnosis using formalin-fixed paraffin-embedded (FFPE) tissues remains essential for the prognostic and therapeutic management of cancer patients. Standardisation of the pre-analytical tissue processing is required to meet requests from both clinicians and patients and to provide immunophenotypic and gene expression data to allow the planning of personalised therapeutic regimens. A proper preservation of the tissue components is pivotal for studies potentially useful to the patients, and recent improvements in the protocols for pre-analysis processing of pathological tissues aim to better preserve cellular details and to conserve antigens and nucleic acid sequences. Amongst the multiple steps involved, the Cold Ischemia time (interval between surgical intervention and transfer to pathology laboratories for grossing and fixation) demands special attention and Guidelines have been issued for a rapid transfer of surgical specimens to the pathology labs. The time-honoured protocol for sending tissues to the pathology department is to immerse the specimen in formalin. This protocol is effective for small biopsies (<2 cm in size) but involves several drawbacks for large specimens. The alternative method of vacuum sealing and cooling (VSC) the specimens represents a well accepted and environmentally safe procedure that overcomes the many drawbacks linked to transfer in formalin. Moreover, RNA is notoriously poorly preserved in FFPE tissue. To overcome this problem, a step forward is represented by our observation that the temperature of fixation is a critical issue and that tissue fixation in cold formalin (at 4 °C) allows a better preservation of nucleic acid sequences.

## Developments of SOP’s and QC for DNA and RNA extraction quantification and quality analysis in molecular archive tissue diagnostics, Peter Riegman

Many parameters could influence while isolating DNA and RNA from tissues during the pre-analytical phase, affecting comparative test results. The derivatives need to be of comparable quality as well as fit for purpose; however, standardisation is not always practical or feasible in a routine hospital setting. In particular, the conditions before and during surgery can have a major influence on samples originating from surgical specimens as well as the duration and temperature during the cold ischemic time (transport).

The freezing method can have an influence on the morphology, which is needed to relate the results of the test to the diagnosis. Formalin fixation and paraffin embedding have influence on DNA and RNA quality. In particular, the routine setting in one laboratory can have a variety of fixation times in daily practice. All the described pre-analytical variations could disturb the results of molecular tests.

To enable the introduction of more sophisticated tests based on tissue samples, making use of sensitive techniques in diagnostics and better opportunities in medical research such variations need to be eliminated to an acceptable level. To control such variations, procedures must be standardised, avoided or recorded. After sample acquisition for frozen tissue, the procedures can be standardised. For FFPE tissues, new solutions have emerged to gain control after acquisition of the material. However before acquisition the only solution is recording, which means that the different conditions of influence during the surgical procedure must be accessible as metadata to biobank personnel and used for result analysis or cohort selection.

## Next-Generation sequencing in diagnostics and clinical research, Gerald Hoefler

Data obtained from analysing the tumour genome by using next-generation sequencing (NGS) technologies have revolutionised our understanding of tumour biology and paved the way to personalised medicine in oncology. In addition, it has also led to completely new concepts in microbiology, allowing, for example, pathogen detection and identification with enormous sensitivity and specificity.

We know now that typical tumours contain two to eight ‘driver gene’ mutations that convey a growth advantage and up to 100 ‘passenger gene’ mutations. Tumour development starts with a gatekeeping mutation that is followed by series of clonal tumour cell expansions that are associated with driver gene mutations providing additional growth advantage. Astonishingly, more than 50% of passenger mutations are thought to occur at the pre-neoplastic phase, leading to the concept that a large proportion of these mutations are random events present in a cell before the initiating event in tumourigenesis. Of further note, almost all mutations of metastatic cancer are already present in the primary tumour. Detecting shedded tumour cells in peripheral blood and especially shedded tumour DNA in serum is likely to become an important test to monitor tumour patients and to design optimal therapeutic approaches. The number of ‘druggable’ mutations has greatly increased in the last few years. For the majority of malignant tumours, it is now obligatory to know the mutational status of target genes before targeted treatment can start. The enormous potential of NGS is now finding its way into daily diagnostic practice in molecular pathology as well as the clinic.

## Proteomics SOPs in archive tissues, Karl-Friedrich Becker

FFPE samples have been used all over the world for the past few decades to distinguish diseased from normal tissues. Because of its cross-linking effects, it is often believed that formalin fixation of routinely processed tissues in the clinic prevents protein profiling.

Recently, major efforts by a number of research groups have led to the development of protocols feasible for the extraction of full-length immuno-reactive proteins or peptides from FFPE tissues. As a consequence, the scientific community now accepts that proteins or peptides extracted from FFPE tissue are suitable for downstream proteomic analysis. Currently, standard operating procedures and technical specifications for the specific steps necessary to extract full-length proteins or peptides from FFPE tissues are being developed.

## SPECTAcolor—the new EORTC screening platform for molecularly driven trials in colorectal cancer, Gunnar Folprecht

With the increasing knowledge on the patho-biological background of colorectal cancer, several subgroups can be distinguished within this cancer. In addition, some molecular markers could be identified to predict resistance to treatment. Although the number of hypotheses and potentially effective drugs has increased, it has become more difficult to screen patients for the molecularly defined trials.

The SPECTACOLOR (Screening Patients for Efficient Clinical Trial Access in COLORectal cancer) study is a new programme of the European Organisation for Research and Treatment of Cancer (EORTC) currently including nearly 20 centres in Europe. Patients at participating centres sign an informed consent that (1) their biological material can be centrally evaluated for molecular markers related to inclusion in clinical trials, (2) additional markers can be determined in the future if new trials are planned and that (3) clinical trials can be offered according to the results of central molecular testing. Patients are followed up for overall survival and intercurrent treatment to optimise the molecular screening.

Recruitment in the first centres started in late September 2013. Currently, more than 400 patients are registered. The number of centres and patients registered per week is rapidly increasing. Besides the primary aim of offering an efficient clinical trial structure, SPECTAcolor is offering opportunities for translational research.

Following SPECTAcolor, more platforms for screening in lung, prostate cancer melanoma and brain tumours are under development.

## Pre-analytical conditions in tissues: the experience of the National Cancer Institute of Genoa, Mauro Truini

The new diagnostic (omics) methods represent one of the most important technological revolutions of this century. These methods allow the analysis of mutations, gene expression and proteins by high throughput technologies representing a key step for targeted therapies. All these techniques require high-quality tissue samples; thus, the lack of control of the pre-analytical phase of tissues processing represents a critical point both for biomarker evaluation and validation and for molecular studies.

Here we present our experience in Genoa. In our Institute, the use of formalin fixative outside the pathology laboratory is now forbidden, and within the laboratory it can be used under aspiration hoods only. Every day biological materials arrive from surgical rooms to our laboratories, within 10 minutes, unfixed; every case is immediately recorded and one pathologist together with one technician samples it either for diagnosis by fixing the sample in 10% buffered formalin at pH 7 for 24 hours or for nitrogen storage in the Biological Resource Centre for research purposes. The time to fixative and the time in fixative are extremely controlled.

Recently, the Italian Society of Pathology and the Italian Society of Oncology issued guidelines with specific recommendations in all molecular pathology fields related to targeted therapy, in accordance with external quality control. In conclusion, standardisation of tissue handling, sample preparation and optimal quality of clinical samples is required to reduce the risk of error in all molecular biology tests.

## Quality of DNA and RNA is critical for any further analyses, Johannes Haybaeck

Quality control has to be taken into consideration at different levels of DNA and RNA extraction. As samples are rapidly degraded by autolysis, they have to be transferred quickly from the operation theatre to the respective pathology department to yield high-quality DNA and RNA. Moreover, large tissue specimens as typically gained from tumour biopsies or even large resection specimens require much longer fixation times than small samples. Nowadays, it is well established that the time from tumour removal to its fixation should be limited to less than 1 hour and according to very recent literature the specimens ideally should be cooled.

Vacuum packing and cooling are interesting alternatives to the potentially carcinogenic formalin fixation for tissue preservation as they allow to a high extent cost-efficient preservation of RNA, DNA and morphology. Cooled and vacuum-packed specimens seem to be a resource for future cell culture work and a preservation tool for stem cells. DNA and RNA can be extracted using different protocols, but independent of the kit used proper positive and negative controls are always mandatory.

Thus, we are moving forward to a new level of preservation to prepare tissue specimens and cells for novel and more sensitive diagnostic as well as research approaches for which RNA and DNA integrity have to be taken more care of. An OECI-driven quality standardisation of used protocols may help to provide the possibility of performing studies with reliable samples from various European Cancer Institutes.

## Development of tissue based biomarkers and circulating biomarkers in cancer: synergies and complementarities, Rares Buiga

Finding new targeted therapies and their corresponding companion diagnostics is a prerequisite for personalised medicine, which seems to be at this moment the only reasonable way to successfully treat cancer. Nowadays, all companion diagnostics approved in clinical practice are tissue-based biomarkers, relying on biopsies or surgical samples taken from the primary tumour [1]. However, cancer is a dynamic condition and his treatment is a moving target. This is due to the heterogeneity of the primary tumour and his intrinsic genetic instability [2]. This leads to a continuous molecular evolution and acquired resistance to treatment [3]. Proper treatment would require serial biopsies, but these are not always feasible or relevant. This imposes the need for more predictive and less invasive diagnostic procedures, like blood-based ‘liquid biopsies’. The most promising techniques are circulating tumour cells (CTC), circulating DNA (ctDNA) and miRNA (from exosomes) which can be repeated over time, allowing a close follow-up of the transformations of the tumour and consequently, better treatment decisions earlier than tissue biopsies would allow [4]. This presentation will show how ‘tissue-based biomarkers’ will continue to lead the process of discovery of new companion diagnostics and how ‘circulating biomarkers’ inferred from them, will permit a personalised fight against cancer.

## DNA extraction phase in the molecular testing procedure: our experience in clinical settings, Maria Dono

The molecular assays currently used to detect the molecular alterations in solid tumours involve the recovery of nucleic acids from diagnostic FFPE tissues or cytological specimens, making all these archived samples an invaluable source for both the discovery and characterisation of the biomarkers. However, the recovery of high-quality nucleic acids from the paraffin block remains challenging as it is well known that tissue fixation with formalin adversely affects DNA quality. First, the protein–protein and protein–DNA cross linkage induces chain breaks. As a consequence, DNA is often heavily fragmented and this limits the downstream molecular reaction needed to analyse its mutational status. Second, the fixation process induces chemical modification of DNA, that is, the cytosine deamination which in turn may lead to G > A transition owing to nucleotide misincorporation during PCR amplification. Despite these critical factors for retrieval of DNA from the FFPE samples, the DNA extracted from the majority of FFPE tissues is applicable for molecular tests, mainly PCR based. In addition, the pressing demand for testing has led to its usage as a source of nucleic acid in very small samples such as tiny biopsies and cytological slides. Testing of both these types of specimens (even from stained slides) is feasible and adopted as a clinical practice in our laboratory. Here, our experience in a clinical setting will be discussed.

## The role of genetic studies in the diagnosis of acute myeloid leukemia, Adam Bagg

The diagnosis and classification of myeloid neoplasms requires, in addition to the integration of clinical features, an array of laboratory studies that include morphology, histology, immunophenotyping (both flow cytometry and immunohistochemistry) and genetic analysis. The genetic studies include classical metaphase analysis, fluorescence *in situ* hybridisation (FISH) and an increasing number of molecular approaches. Molecular analysis provides tremendous new tools for diagnosis and classification, and simultaneously helps to unravel the biology of these neoplasms so that patients may benefit from personalised therapeutic approaches. To highlight the relevance of genetic studies in myeloid malignancies, this lecture focuses on contemporary genetic approaches in the evaluation of acute myeloid leukemia, from metaphase cytogenetic analysis, through single gene analysis, to multiplexed approaches including NGS lysis through single story Medcinemia.

## Moving from Sanger to next generation sequencing: our experience in the genetic diagnosis context, Jose Antonio López-Guerrero

With the development of high throughput, NGS technology has revolutionised the molecular diagnosis of human disease and especially cancer. The ability to generate enormous amounts of sequence data in a short time at an affordable cost makes this approach ideal for a wide range of applications from sequencing a group of candidate genes, all coding regions (known as exome sequencing) to the entire human genome. Worldwide efforts to catalogue mutations in multiple cancer types are underway and this is likely to lead to new discoveries that will be translated to new diagnostic, prognostic, and therapeutic targets. NGS is now growing to the point where it is being considered by many laboratories for routine diagnostic use. The sensitivity, speed, and reduced cost per sample make it a highly attractive platform compared with other sequencing modalities.

The Fundación Instituto Valenciano de Oncología (IVO) is a non-profit-making organisation devoted to the fight against cancer in terms of prevention, treatment, and research. Through its Laboratory of Molecular Biology, we provide molecular and genetic support to the clinical management of cancer patients, sequencing constituting the keystone of our activities. This lecture provides a view of how our laboratory has adapted its procedures to the successive incorporation of different sequencing methodologies including sample processing, laboratory infrastructure, personnel training, software, genetic diagnostic kits for germline and somatic mutations, costs and new opportunities.

## NGS for molecular tests in colorectal cancer: which sensitivity is required for clinical decision making?, Simonetta Zupo

Targeted therapies have been developed for cancer patients who bear specific somatic mutations in specific genes. Thus, the presence of mutations in RAS, EGFR, BRAF, CKIT, PI3K, PDGFR genes are considered predictive biomarkers and routinely detected in a clinical setting. NGS technology has expanded beyond research, becoming an important tool in current molecular diagnostics. However, the implementation of this technology in a clinical laboratory environment still has to be validated. One of the main issues for the validation of NGS technologies in cancer-predictive medicine regards the clinical analytical sensitivity that NGS mutational test would reach to deliver a correct predictive information to physicians [5]. Colorectal cancer represents an exhaustive example where the presence of somatic mutations in specific genes identifies the patients resistant to anti-EGFR therapy. In this context, the presence of a few mutated subclones detectable only by high-sensitivity NGS could influence the response of the colorectal tumour to target therapy in two opposite ways: (1) they do not affect the response of the tumour to anti EGFR therapy suggesting that the detection by high sensitivity method could exclude potentially responsive patients from anti EGFR therapy and (2) they negatively affect the response to anti-EGFR therapy suggesting that higher sensitivity-genotyping method may enhance the predictive value of these molecular markers.

A correct detection limit of NGS for clinical application will improve the selection of patients who might benefit from treatments involving anti-EGFR.

## Day hospital and pharmacy: partners for the patient’s benefit, François Lemare

Anti-cancer drug compounding is a highly technical activity that requires a high level of quality to provide a sterile preparation be able to meet pharmaceutical requirements in terms of drug stability and sterility. From this point of view, the centralisation of anti-cancer drug preparation under the direct supervision of the pharmacy staff is required by the OECI as by many other institutions or regulatory agencies to guarantee drug quality. However, the definition of quality always refers to an observatory point. Attempting to satisfy the requirements of nurses, patients, oncologists, pharmacists and economic directors simultaneously may seem impossible. Therefore, the main objectives are summarised as 5Rs: *R*ight drug to the *R*ight patient at the *R*ight dosage in the *R*ight schedule in the *R*ight condition.

For this purpose, collaboration with outpatients is crucial in this organisation. Both the pharmacy and outpatient ward are interdependent. The quality of the service requires definition of the objectives for both sides as well as a continuous communication between the teams. For instance, the duration of one preparation can appear unimportant when it is compounded at the bedside but simultaneous compounding of 20 preparations may take time. On the other hand, even if all preparations have to be compounded, a respectful dispensation of the schedules of administration allows a better organisation of the outpatient ward.

Finally, defining the priorities of each of the actors leads to a significant improvement in the organisation to the benefit of the patient and the teams.

## Empowering patients’ organisations to overcome inequalities in cancer care, Francesco de Lorenzo

Despite improvements made in cancer care, there is still unequal access to services, treatments, social care and follow-up for patients and survivors. At Member State level, there are great variations in the implementation of national policies, plans or strategies to tackle cancer. The unbearable inequalities existing in cancer care in Europe are exemplified by the differences among the average cancer expenditures per citizen in the EU (102 €), compared with Bulgaria (16 €), Romania (20 €), Poland (37 €), Portugal (53 €), UK (85 €), Spain (94 €) France (110 €), Italy (114 €) and Germany (182 €). Cancer care expenditures are directly related to survivorship: EUROCARE 5 study clearly demonstrates how 5-year survivorship rates are from 20–40% lower in Eastern European countries, compared with Western European ones.

To reduce inequalities, the National cancer plans should be harmonised through the adoption of cancer care guidelines ensuring minimum requirements for each European cancer patient. Within EPAAC, the ECPC contributed to the establishment of a European Guide for Quality National Cancer Control Programmes. The CANCON project, built on the EPAAC successes, will enlarge the existing guidelines, including survivorship, rehabilitation and a new Member State platform for continuous collaboration. ECPC believes that inequalities in cancer care can be finally eradicated only through the establishment of a European Cancer Plan. A first step would be to establish European care models based on the studies carried on by EUROCHIP on breast cancer, introducing minimum care standards and affordable quality and equitable care at the EU level. The expected results from the BenchCan project, laying down the basis to compare cancer care among Member States, could be of a great support to the establishment of a European Cancer Plan.

## Implementing large scale fast track diagnostics in a comprehensive cancer centre, Nicolette Goedbloed, WH van Harten

**Background:** In general, patients suspected to have cancer visit the hospital multiple times before the diagnosis is completed. Fast growing patient numbers and increasing process complexity has led to diminished service levels. To decrease the amount of visits, fast track diagnostics have been implemented for 18 cancer types. We report on the process of redesign, implementation and first results.

**Methods:** Throughput time (first visit to diagnosis conversation) was measured before redesigning and again after implementation. With an analysis, the process was redesigned involving multidisciplinary teams. In an eclectic approach, elements from lean management, theory of constraints and mathematical analysis were used to organise slots for MRI, CT, PET and echography, without detriment in scheduling for other categories. Post-measurement was performed after three and six months.

**Results:** In pre-measurement, access time was estimated as 10–15 workdays, and mean throughput time 13.3 workdays. It proved possible to design the diagnostic process of 18 tumours as fast track of which seven were a ‘one stop shop’ (diagnosis completed in one visit). Involvement of clinical and board leadership, massive communication efforts, commitment of physicians to reschedule their activities proved decisive. After three and six months the mean access time was 7.2 (median 6) respective 7.7 workdays (median 6) and mean throughput time was 2.4 (median 0) respective 2.3 workdays (median 0).

**Conclusions:** It proved possible to redesign and implement fast track diagnostics for 18 cancer types. Throughput time and access time were considerably shortened. Involvement of physicians in reorganising their work was a crucial success factor [6, 47].

## Importance of a psycho-oncology team, Darius Razavi

The important prevalence of psychosocial problems and psychiatric disturbances that have been reported in oncology underlines the need for comprehensive psychosocial support of cancer patients and their families. Psychosocial interventions designed for this purpose should be divided into five categories: prevention, early detection, restoration, support and palliation. First, preventive interventions are designed to avoid the development of predictable morbidity secondary to treatment and/or disease. Second, early detection of patient’s needs or problems refers to the assumption that early interventions could have therapeutic results superior to those of delayed support, both for quality of life and survival. Third, restorative interventions refer to actions used when a cure is likely, the aim being the control or elimination of residual cancer disability. Fourth, supportive rehabilitation is planned to lessen disability related to chronic disease characterised by numerous cancer illness remission, progression and active treatment. Fifth, palliation is required when curative treatments are likely to be no more effective, and when maintaining or improving comfort becomes the main goal. Psychosocial interventions are often multidisciplinary with a variety of content. The content of psychosocial interventions range from information and education to more sophisticated support programmes including directive (behavioural or cognitive) therapies, or non-directive (dynamic or supportive) therapies. These interventions may be combined with the prescription of pharmacological, physical, speech or occupational therapies, especially in rehabilitation programmes. Descriptive and empirical studies available today are suggesting the need for truly multidisciplinary and strongly coordinated interventions to be significantly effective and to benefit to cancer patients and families. These concepts will be presented and discussed on the basis of the experience of the Jules Bordet Institute.

## GRTPs: good radiation therapy practices, József Lövey

Quality management gains are increasing in importance in all areas of health care. Radiotherapy has always been at the head of these processes as it has been proven that quality of care and outcome are closely related. Radiotherapy QA is more complex than checking the machines. There are pyramid like levels of radiotherapy QA and most of these can be effectively quality assured. Of course a good physical QA and maintenance programmes of machines is the first. The next is an applied level: physical QA of treatment planning (measurements + planning process) and the medical part of it; mostly the region of interest definition and delineation. The next level is the integrated medical QA of the treatments that involves indication, imaging, MDT decision and performing radiation treatment itself including side effect monitoring and registration of misses and near misses and ensuring the appropriate support during radiotherapy and rehabilitation afterwards.

A level higher is the assurance of the right place and patient flow of radiotherapy in the context of the whole hospital; to organise the patient admission and then optimising the patient pathway inside radiation oncology with special attention to the waiting times; to register the technical and clinical needs; developing adequate consensus between radiation oncology and the partner disciplines (MDTs); managing the human resources (skill mix) and capacity of technical facilities.

The next level is the strategic management of radiation services. Planning of the development of the centre to ensure smooth working in a view of 5–10 years ensuring quality improvement in all areas mentioned above. An important and different part of this strategic management is the attention to the economic environment and reimbursement policies.

## Evidence based surgical and gynaecologic oncology care in specialised cancer centres—benefits for patients, Patriciu Achimas Cadariu

Surgery was the first and only treatment for cancer from a historical point of view. Surgeons have a central role in treatments and research, leading the diagnostic and treatment pathways for most cancers from counselling patients about their diagnosis through to surgery and aftercare [7–9]. The evolution of the genome within a dynamically changing adaptive landscape leads to the outcome of genomic and phenotypic heterogeneity, which both negatively affect the ability of targeted therapies to exert cancer control. Taking into account the dramatic escalation of costs in the management of malignant neoplasia, surgical subspecialties hold an important role in curing premalignant diseases and as part of the multidisciplinary approach in the treatment of malignant ones [24–29].

We present the most significant evidence as far as surgical oncology and gynaecologic oncology into the treatment of premalignant [10–23] as well as malignant diseases [30–42] are concerned in organ centres, cancer centres and comprehensive cancer centres, emphasising interdisciplinary collaboration, guideline-compliant treatment, and expertise of the main treatment partners. Indicators reflecting pre-treatment and postoperative case presentations in multidisciplinary team meetings, psycho-oncologic care as well as social service are also discussed always focused on reducing mortality due to cancer and improving patient quality of life. We further discuss the importance of subspecialist training [44, 48], the current status of it in different EU countries [45, 46], in the frame of the Union of European Medical Specialists (UEMS) regulations [43], the final goal being to provide evidence of expertise in the subject at a level that would be acceptable in all European Countries.

## Fusion imaging targeting for biopsy and therapy of lesions with poor US conspicuity, Gian Andrea Rollandi, Francesco Paparo

Personalised and tailored therapies play an increasingly critical role in cancer care. Multimodality fusion imaging (MMFI) is the process of aligning and juxtaposing images obtained by two or more imaging modalities; it allows for combining morphological, functional and metabolic information in a single fused image. Spatial co-registration is necessary to ensure that the pixels from the various datasets represent the same volume with acceptable precision [49]. Recent studies have demonstrated that the multimodal co-registration, synchronised navigation and combined interpretation are more valuable than the individual, separate assessment of different diagnostic techniques [50]. MMFI may involve computed tomography (CT), magnetic resonance imaging (MRI), positron emission tomography (PET) and real-time ultrasound (US). From a diagnostic point of view, MMFI is able to improve the molecular profiling of tissues, providing their comprehensive and accurate non-invasive characterisation. From an interventional point of view, MMFI can be employed to successfully assist biopsy and ablation procedures of technically challenging targets [51, 52]. For example, some PET-positive lesions may have little or no correlative US and/or CT findings (i.e. low conspicuity on morphological imaging). As it is not possible to perform biopsy under PET guidance alone, owing to intrinsic technical limitations, the metabolic information of PET has to be integrated into a CT- or US-guided biopsy procedure. The latter technique provides the real-time monitoring of the needle track towards the target lesion, avoiding the use of ionising radiation. MMFI between real-time US and PET/CT has been recently proposed for guiding the biopsy procedures of PET-positive lesions with absent or low conspicuity on US images, and this new integrated approach is giving encouraging results [52].

## Introducing personalised medicine in eastern and western European settings, Marco A Pierotti

The ultimate goal of post-genomic medicine—also called ‘4 P medicine’ is to provide the right drug to the right person at the right time. Integrating the molecular and the classical histopathology classifications of tumours is a consequence of the accepted concept of cancer as a somatic genetic disease and the new molecular characterisation of tumours.

As a consequence, the same tumour histotype can be subdivided into different subtypes according to the gene expression profile more significantly associated with survival than the traditional ones. Moreover, by highlighting how several pathways comprising numerous interacting genes are deregulated in cancers, molecular classification has also challenged the paradigm according to which different cancers are different diseases. This concept must be coupled with the awareness that the same molecular defects are characteristic for different tumour histotypes, like, for example, EGFR mutations for lung and colon, and PI3K mutations for colon and breast carcinomas.

This finding prompts the switch from the traditional idea of drugs—as compounds with a specific and preferential activity on tumour cells—to knowledge-based targeted or personalised therapy.

By increasing survival from 15 months to more than 15 years from diagnosis in chronic myeloid leukemia, treatment with Imatinib (Gleevec), was the first successful targeted therapy. Several other ‘druggable’ targets have been identified, thus supporting the paradigm of personalised medicine in cancer care. The financial sustainability of targeted therapies was questioned because of cost/benefit considerations. In fact, it emerged that the story of Imatinib/CML was perhaps an exception.

The lesson we have learned is that, while chronicisation is a winning strategy in the treatment of diabetes or cardiovascular diseases, it fails in the treatment of cancer because of the genetic instability of cancer cells. To be successful, targeted therapies should eradicate tumour cells and, to achieve this goal, we will probably need to combine different types of therapies including targeted therapy and agents modulating the immune system. We might even need to modify the macro (metabolism) or micro (stroma) tumour environment.

## Introduction of personalised therapy in Romania, Tudor E Ciuleanu

As of May 2014, the recognised ‘official’ standard of care in cancer is still represented by the 2009 edition of the ESMO guidelines. Cancer treatment is free, as the National Programme of Oncology covers the budget for all cytotoxic agents and targeted therapy. However, reimbursement for several expensive drugs for solid tumours, such as bevacizumab, cetuximab, erlotinib, imatinib, pemetrexed, sorafenib, sunitinib, trastuzumab, is individually approved by a centralised commission with monthly reunions. Analysis of biomarkers is not covered by the insurance companies or the state budget. Some of them are sponsored by the pharmaceutical industry (such as HER2 FISH testing, mutational status for EGFR or KRAS). All the new drugs registered in Europe by a common procedure by the European Medicines Agency are concomitantly authorised for medical use in Romania. However, no new drugs (such as abiraterone, afatinib, aflibercept, axitinib, cabazitaxel, cabozantinib, catumaxomab, crizotinib, dabrafenib, degarelix, denosumab, enzalutamide, eribulin, everolimus, gefitinib, ipilimumab, lapatinib, panitumumab, pazopanib, pertuzumab, regorafenib, sipuleucel, trabectedin,trastuzumab emtansine,vandetanib, vemurafenib, vinflunine, vismodegib) and no new indications (such as 1st line TKIs or maintenance treatment in NSCLC, trastuzumab in early breast cancer or advanced gastric cancer, bevacizumab in gynaecological cancers, sunitinib in neuroendocrine tumours) have been being accepted for reimbursement since 2008.

On the other hand, clinical research is rapidly growing and Romanian centres demonstrate a high recruitment rate in pivotal trials, despite initial delays due to a slow approval of the studies by the competent authorities.

## Patients’ perspectives in the delivery of cancer care, Paolo De Paoli

Patients can improve care by collaborating with health care workers to design new organisation and educational systems. Patient-centred care requires that patients, health care workers and health care organisations acquire a specific set of competences. In our Institute, CRO Aviano, this strategy is based on a specific PE Programme and group that is multidisciplinary, non-hierarchical that actively involves patients. Furthermore, to provide a stable support and strategy nationwide on PE, we established a research programme aiming at assessing PE programmes in Italian CCCs; we were interested in four key points: (1) presence of institutional policies for PE; (2) activities routinely part of PE; (3) *involvement of patients* representatives; and (4) *role of health care workers*. Our data show that institutional PE policies are active in the majority of Italian CCCs based on patients’ needs (in particular classes and patients’ handout). Caregivers are involved in 73% of the Italian institutions. Health care workers receive a specific training and have an active role in the organisation of PE programmes. Our data suggest that the role of patients in oncology care is increasing and their needs are taken into consideration. However, our survey indicates that changing the mind set from a provider-focus to a patient-focus is still a major barrier to patient-centred care. To overcome this barrier, cancer centres and patients representatives should design an institutional approach to improve patient-centred care. In addition, tracking and monitoring progresses in PE programmes requires affordable measures to assess whether PE programmes are really improving outcomes.

## Combating inequalities in a high tech oncology service environment, George Weiner

Dr. Weiner serves as Vice President/President Elect of the Association of American Cancer Institutes. He reviewed how cancer centres in the United States are organised and accredited. United States has a number of organisations that support and provide accreditation for various missions of the US cancer centres. These include the National Cancer Institute, the Association of American Cancer Institutes, the American College of Surgeons Committee on Cancer, and regional Comprehensive Cancer Control organisations based in various states. Some of these efforts focus on the quality of clinical care, others on research and others on cancer prevention and early detection. He reviewed the various roles of these organisations, how they impact on the success of academic cancer centres in the United States, and how improved coordination between these organisations would benefit the US Cancer Centres.

## Quality perspective 2015–2020: update of accreditation and designation system, Mahasti Saghatchian

To improve the quality of care in Cancer Centres (CC) and designate Comprehensive Cancer Centres (CCC), the Organisation for European Cancer lnstitutes (OECI) launched in 2002 a project for Accreditation and Designation (A&D) of European Cancer centres. The programme was officially launched in 2008.

The accreditation process starts with a preliminary designation followed by a six-month self-assessment and a peer-review visit by four auditors from different specialties. Once reviewed, the OECI delivers a report identifying the opportunities and threats for the centre and recommendations for improvement. If approved, the OECl gives the accreditation and a final designation as a ‘cancer unit’, ‘clinical care centre’ (cc); ‘cancer research centre’ or ‘ccc’ (comprehensive cancer centre). Data collected during self-assessment and peer-review from the participating centres are combined in a database for reporting and comparative analysis.

Assessment of the first centres showed that volumes of various functions and activities dedicated to care, research and education vary widely among centres. As the final goal of the OECI is to improve quality of care by integration of high-quality cancer research, the OECI is currently developing a benchmarking project. The general objective of this project is to benchmark comprehensive cancer care and yield best practice examples in a way that contributes to improving the quality of interdisciplinary patient treatment through research. Finally the A&D Programme is in the process of reviewing the standards and a new questionnaire will be developed for the next round.

## Biomarkers, genetic testing, biobanks and pain management

Four additional speakers at the OECI Oncology Days meeting included Bogdan Fetica from the Oncology Institute ‘Prof. Dr. Ion Chircuţă’, Cluj-Napoca in Romania, Jean-Christope Sabourin of the Rouen University Hospital in France, Paul Hofman of the University of Nice Sophia Antipolic in France and Sopie Laurent from Institut de Cancérologie Gustave Roussy, also in France.

Bogdan Fetica discussed biomarkers in breast cancer; in particular, he focussed on the growth in the number of immunohistochemical markers used in prognostic and therapeutic decisions. He also stressed the importance of standardisation and validation of immunohistochemistry and ISH techniques in Romania and internationally.

Jean-Christope Sabourin outlined the development of government funded genetic testing in France to ensure that all cancer patients are offered this service, regardless of their region of origin. Since 2006, 28 regional molecular laboratories have been being funded through the INCa (Institut National du Cancer, or French National Cancer Institute). As the cost of targeted therapies far outweighs the cost of molecular testing, undergoing such screening prior to treatment is an economically sound approach. The French scheme has already demonstrated its success with the entire French population with either metastatic colorectal carcinoma (18,000 patients) or advanced or metastatic broncho-pulmonary adenocarcinoma (22,000 patients) screened in 2012.

Paul Hofman discussed the use of cancer biobanks based in hospitals. He looked at some of the considerations for establishing biobanks and some of the challenges in sharing information between biobanks and between a biobank and researcher.

Sophie Laurent focussed her talk on the assessment and treatment of pain for Gustave Roussy’s patients. An institutional strategy started in 2009 adopted a three-pronged approach involving patients, health care professionals and the Institute. Five years later, pain assessment and traceability in patient charts had improved from 45–98%.
